# Empagliflozin in resistant hypertension and heart failure with preserved ejection fraction: the EMPEROR-Preserved trial

**DOI:** 10.1093/eurheartj/ehae938

**Published:** 2025-03-04

**Authors:** Michael Böhm, Javed Butler, Andrew Coats, Lucas Lauder, Felix Mahfoud, Gerasimos Filippatos, João Pedro Ferreira, Stuart J Pocock, Martina Brueckmann, Sibylle J Hauske, Elke Schueler, Christoph Wanner, Subodh Verma, Faiez Zannad, Milton Packer, Stefan D Anker

**Affiliations:** Klinik für Innere Medizin III, Universitätsklinikum des Saarlandes, Saarland University, Kirrberger Str.1, Homburg/Saar 66421, Germany; Cape Heart Institute, Cape Town, South Africa; Baylor Scott and White Research Institute, Dallas, TX, USA; Department of Medicine, University of Mississippi, Jackson, MS, USA; Heart Research Institute, Sydney, Australia; Klinik für Innere Medizin III, Universitätsklinikum des Saarlandes, Saarland University, Kirrberger Str.1, Homburg/Saar 66421, Germany; Cape Heart Institute, Cape Town, South Africa; Department of Cardiology, University Heart Center, University Hospital Basel, Basel, Switzerland; Cardiovascular Research Institute Basel (CRIB), University Heart Center, University Hospital Basel, Basel, Switzerland; Klinik für Innere Medizin III, Universitätsklinikum des Saarlandes, Saarland University, Kirrberger Str.1, Homburg/Saar 66421, Germany; Cape Heart Institute, Cape Town, South Africa; Department of Cardiology, University Heart Center, University Hospital Basel, Basel, Switzerland; Cardiovascular Research Institute Basel (CRIB), University Heart Center, University Hospital Basel, Basel, Switzerland; School of Medicine, National and Kapodistrian University of Athens, Athens University Hospital Attikon, 1 Rimini St, Athens 12462, Greece; Centre d'Investigation Clinique- Plurithématique Inserm CIC-P 1433, Université de Lorraine, Nancy, France; Inserm U1116, CHRU Nancy Brabois, F-CRIN INI-CRCT (Cardiovascular and Renal Clinical Trialists), Nancy, France; Cardiovascular R&D Centre—UnIC@RISE, Department of Physiology and Cardiothoracic Surgery, Faculty of Medicine, University of Porto, Porto, Portugal; Department of Medical Statistics, London School of Hygiene & Tropical Medicine, London, UK; Therapeutic Area of Cardiorenal and Metabolic Medicine, Boehringer Ingelheim International, Binger Str. 173, Ingelheim 55218, Germany; First Department of Medicine, Faculty of Medicine Mannheim, University of Heidelberg, Mannheim, Germany; Therapeutic Area of Cardiorenal and Metabolic Medicine, Boehringer Ingelheim International, Binger Str. 173, Ingelheim 55218, Germany; Fifth Department of Medicine, Faculty of Medicine Mannheim, University of Heidelberg, Mannheim, Germany; mainanalytics GmbH, Sulzbach, Otto-Volger-Str. 3c, Sulzbach/Taunus 65843, Germany; Department of Clinical Research and Epidemiology, Comprehensive Heart Failure Center (CHFC), Am Schwarzenberg 15, Würzburg 97078, Germany; Division of Cardiac Surgery, St Michael’s Hospital, Toronto, Ontario, Canada; Department of Surgery, University of Toronto, Toronto, Ontario, Canada M5T 1P5; Department Pharmacology and Toxicology, University of Toronto, Toronto, Ontario, Canada M5G 2C8; Institut Lorrain du Coeur et des Vaisseaux, Nancy, France; Department of Cardiovascular Science, Baylor University Medical Center, 3500 Gaston Ave, Dallas, TX 75246, USA; Faculty of Medicine, National Heart and Lung Institute, Imperial College, Exhibition Road, London SW7 2AZ, UK; Department of Cardiology (CVK) of German Heart Center Charité, Berlin, Germany; German Centre for Cardiovascular Research (DZHK) partner site Berlin, Charité Universitätsmedizin Berlin, Berlin, Germany

**Keywords:** Empagliflozin, Blood pressure categories, Resistant hypertension, Controlled hypertension, Uncontrolled hypertension, Cardiovascular outcomes, Kidney outcomes

## Abstract

**Background and Aims:**

Hypertension has a high prevalence in heart failure with preserved ejection fraction (HFpEF), which can be controlled, uncontrolled, or even resistant. The effects of empagliflozin on systolic blood pressure (SBP), time in target range, incidence of hypertensive urgencies, and studied cardiovascular and renal outcomes in different hypertension categories and after treatment with empagliflozin in the EMPEROR-Preserved trial were explored.

**Methods:**

A total of 5533 patients were studied and the population was separated into resistant (resHTN), uncontrolled (uctrHTN), and controlled (ctrHTN) hypertension. The effect of SBP on outcomes and treatment effects of empagliflozin were explored. Analyses were done with Cox regression analyses adjusted for demographic and clinical confounders and with a mixed model for repeated measures.

**Results:**

Empagliflozin reduced SBP in resHTN slightly more than in the other categories in the first weeks, while thereafter there were no significant differences. The modest reduction in SBP resulted in a moderate increase in time at target and reduced hypertensive urgencies. The primary endpoint was more prevalent in resHTN (*P* = .0358), but the treatment effect of empagliflozin on the primary endpoint was similar in resHTN, uctrHTN, and ctrHTN (*P* for interaction = .92) as was the improvement of the estimated glomerular filtration rate slope (*P* for interaction = .95) and change in quality of life by empagliflozin.

**Conclusions:**

In HFpEF, the prevalence of resHTN is high and is associated with frequently higher outcome rates compared with ctrHTN and uctrHTN. The treatment effect was not modified by hypertension categories. This indicates that in HFpEF, moderate modifications of blood pressure do not affect overall outcomes and treatment effects of empagliflozin.

## Structured Graphical Abstract

**Structured Graphical Abstract:**
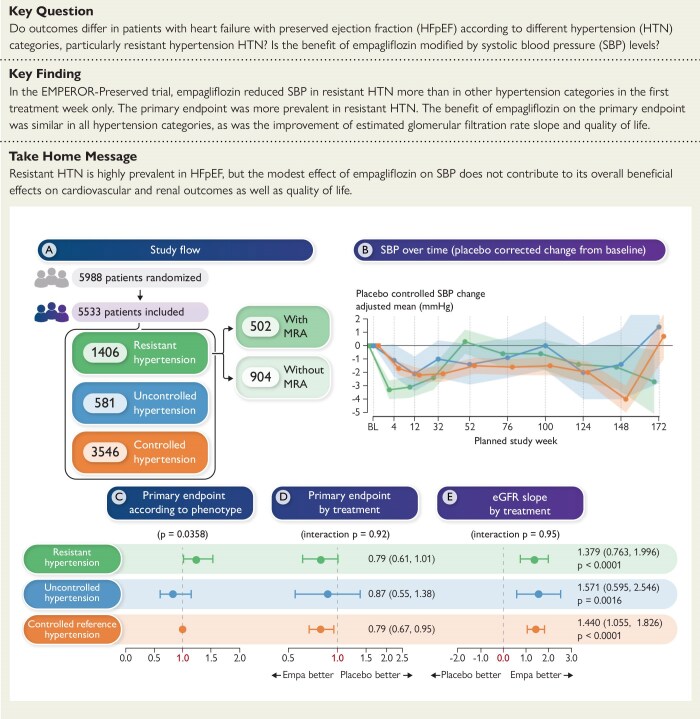
Study flow (*A*), effect of empagliflozin on systolic blood pressure over time (*B*), incidence of primary endpoint within placebo (*C*), effect of empagliflozin on the primary endpoint (*D*), and the effect on the estimated glomerular filtration rate slope (*E*) in patients with resistant hypertension, uncontrolled hypertension, and controlled hypertension. eGFR, estimated glomerular filtration rate; HFpEF, heart failure with preserved ejection fraction; HTN, hypertension; MRA, mineralocorticoid receptor antagonist; SBP, systolic blood pressure.

**See the editorial comment for this article ‘SGLT2 inhibitors: not for hypertension but exceedingly useful in hypertension’, by F.H. Messerli, https://doi.org10.1093/eurheartj/ehae751.**

## Introduction

Hypertension is the most prevalent risk factor for incident heart failure.^1^ In heart failure with preserved ejection fraction (HFpEF), the prevalence of previous hypertension ranges between 55% and 90%^2,3^ and is higher compared with patients with heart failure with reduced ejection fraction (HFrEF).^4^ Blood pressure (BP) control in hypertension is a powerful prevention tool against HFpEF.^5–8^ In overt HFpEF, hypertension is the most prevalent comorbidity linked to worse outcomes.^4,5^ Resistant hypertension (resHTN) is defined as uncontrolled and persistently elevated BP despite treatment with at least three antihypertensive drugs, including an inhibitor of the renin–angiotensin system, a calcium channel blocker, and a diuretic in adequate doses.^5–9^ Resistant hypertension associates with higher rates of cardiovascular outcomes and a higher prevalence of comorbidities compared with patients with treated and controlled hypertension.^10–12^ While in patients with HFpEF, sodium-glucose cotransporter 2 (SGLT2) inhibition with dapagliflozin and empagliflozin is established to reduce cardiovascular death (CVD) and heart failure hospitalization (HFH) as well as to protect kidneys and improve quality of life,^13,14^ the role of BP reduction and modification of the treatment effects of empagliflozin in the presence of controlled hypertension (ctrHTN), uncontrolled hypertension (uctrHTN), and resHTN is not well understood. As supported by a recent registry, the population with resHTN accounts for ∼17% of HFpEF and is lower in HFrEF (10%).^15^ In this analysis of the EMPEROR-Preserved trial, we explored the association to outcome by categories of hypertension with ctrHTN [systolic BP (SBP) 110–140 mmHg, irrespective of number of antihypertensive drugs], uctrHTN (SBP > 140 mmHg and less than three antihypertensive drugs), and resHTN (SBP > 140 mmHg on three or more drug classes, one being a diuretic). Mineralocorticoid receptor antagonists (MRAs) are recommended as fourth-line agents for the treatment of resHTN. Therefore, the latter group was further subdivided into those who are not treated in resHTN with an MRA and those who are treated with an MRA but remained still uncontrolled despite MRA treatment (sometimes referred to as ‘refractory’ hypertension).^5^ The following outcomes were analysed in the different hypertension categories: the effect of empagliflozin on SBP, on the time in target SBP range, on incident hypertensive urgencies as well the treatment effect of empagliflozin in the different categories on cardiovascular outcomes, kidney dysfunction, and quality of life. We hypothesized that empagliflozin has moderate effects on SBP and time in target range as well as a consistent treatment effect in patients with HFpEF irrespective of the hypertension categories.

## Methods

### Study design

The design and results of the EMPEROR-Preserved trial have been published previously.^13,16^ The ethics committees of each of the participating institutions approved the protocol. All patients gave written informed consent. The registration of the identifier at ClinicalTrials.gov is NCT03057951. Patients with heart failure and ejection fraction > 40% were randomized in a double-blind 1:1 fashion to receive either placebo or empagliflozin 10 mg in addition to the usual drug therapy as defined at the discretion of the treating physicians. Patients with SBP > 180 mmHg, symptomatic hypotension, and/or SBP < 100 mmHg at randomization were excluded, and patients with SBP > 150 and <180 mmHg at randomization should be receiving at least three antihypertensive drugs. If eligibility criteria were fulfilled, patients underwent BP measurements in a sitting position after 5 min of rest at the screening and follow-up visits. At screening, the mean of three BP measurements was used to determine eligibility. Blood pressure was taken at each visit similarly by a standard manometer with an appropriate cuff size at the same arm. Patients were assessed at all study visits for major outcomes, vital signs, and creatinine-based estimated glomerular filtration rate (eGFR) according to the Chronic Kidney Disease Epidemiology Collaboration formula. Changes in medications or clinical status that reflected changes in the course of heart failure were recorded and documented. All randomized patients were followed up according to the intention-to-treat principle. The trial conforms to the principles of the Declaration of Helsinki.

### Systolic blood pressure analysis

**Figure 1:**
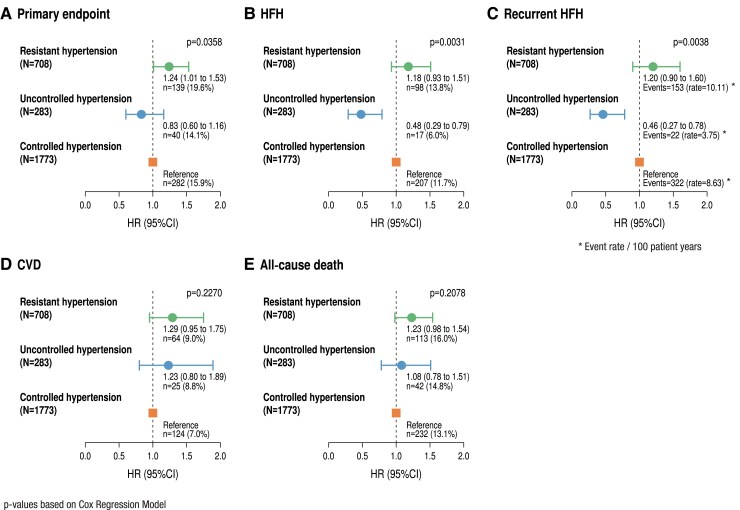
Outcomes according to hypertension categories on placebo. Hazard ratio for the primary endpoint (*A*), heart failure hospitalization (*B*), recurrent heart failure hospitalization (*C*), cardiovascular death (*D*), and all-cause death (*E*) in resistant hypertension, uncontrolled hypertension compared with controlled hypertension (reference) in patients treated with placebo. *P*-values for hypertension category are derived from Cox regression models and joint frailty model, respectively, adjusting for the competing risk of cardiovascular death (for recurrent heart failure hospitalization; *C*). Cox proportional hazard regression models and the joint frailty model were examined using prespecified covariates of age, sex, geographical region, diabetes status at baseline, left ventricular ejection fraction, and estimated glomerular filtration rate at baseline and hypertension category. CI, confidence interval; CVD, cardiovascular death; HFH, heart failure hospitalization; HR, hazard ratio

Patients were categorized by the European Society of Cardiology (ESC)^17^ and the European Society of Hypertension (ESH)^7^ guidelines. Resistant hypertension was defined as SBP > 140 mmHg on three or more antihypertensive drug classes, one being a diuretic. The comparator categories were ctrHTN (110–140 mmHg, irrespective of the number of used medications) and uctrHTN (>140 mmHg) on less than three drug classes. A further analysis was done in patients with resHTN, when they were on an MRA or without an MRA. In patients with HFpEF in clinical trials and registries, U- or J-shaped SBP–risk relationships were observed indicating that low SBP values are also linked to poor outcomes, most likely due to inverse causation.^18,19^ Therefore, patients with baseline SBP < 110 mmHg (*n* = 455) were excluded from this analysis. The subgroup with the inverse risk–SBP relationship has been published before^20^ and showed slightly higher event rates on placebo based on 30 events in these 455 patients. Time in target range and time above range were determined by taking 120–130 mmHg as guideline-directed treatment targets recommended by the European guidelines.^17^ A study flow scheme is depicted in *Figure 1*. We furthermore explored the effect of empagliflozin on hypertension urgencies defined via different criteria such as investigator-reported adverse event based on the following preferred terms ‘hypertensive crisis’, ‘hypertensive emergency’, ‘hypertensive encephalopathy’, ‘hypertensive end-organ damage’, ‘hypertensive urgency’, ‘malignant hypertension’, ‘malignant hypertensive heart disease’, and ‘malignant renal hypertension’ or based on measured BP: SBP > 180 mmHg or diastolic BP (DBP) > 120 mmHg; SBP > 160 mmHg or DBP > 100 mmHg; or a composite based on adverse events and the SBP definitions.

### Outcome measurements

The primary composite endpoint of adjudicated CVD or HFH and the individual components of the composite were analysed as time to first event. The first secondary endpoint was adjudicated total HFH including first and recurrent events. Furthermore, we studied the slope of change from Week 4 in eGFR as the second secondary endpoint, quality of life assessed by the Kansas City Cardiomyopathy Questionnaire Clinical Summary Score (KCCQ-CSS) at Weeks 12, 32, and 52 and all-cause mortality. We explored the influence of hypertension categories on these outcomes, the effect of empagliflozin on SBP and time in or above target range, and the treatment effect of empagliflozin on cardiovascular outcomes in these hypertension categories. Time in range (120–130 mmHg) and time above range (>130 mmHg) were derived based on percentage of days with values in the respective range considering interpolated SBP values from baseline to last SBP measurement on treatment respective occurrence of primary endpoint. The effects of empagliflozin on SBP were determined over 172 weeks.

### Statistical analysis

Baseline characteristics are shown as frequency with percentage mean ± standard deviation or medians with interquartile range. The effect of different hypertension categories on outcomes in the placebo group and the effects of empagliflozin compared with placebo on the time to first event were examined using Cox proportional hazard regression models with prespecified covariates of age, sex, geographical region, diabetes status at baseline, left ventricular ejection fraction, and eGFR at baseline. The first secondary outcome of total (first and recurrent) HFH was evaluated using the joined frailty model that accounted for informative censoring because of CVD. Changes in SBP and KCCQ-CSS were analysed in a mixed model for repeated measures (MMRM). Between-group differences in the slope of eGFR were analysed using a random slope model on on-treatment data. The slope, the joined frailty, and MMRM models included the same covariates as the Cox models. The interaction between hypertension categories and treatment group on the occurrence of the prespecified outcomes was tested using a treatment-by-hypertension category interaction term. All analyses were performed using SAS version 9.4 (SAS Institute, Cary, NC, USA). All *P*-values reported are two sided, and *P* < .05 was considered statistically significant in all cases. No adjustments for multiple testing were made due to the exploratory nature of the study.

## Results

### Patient characteristics

A total of 5988 patients were randomly assigned to receive either empagliflozin (*n* = 2997, 10 mg once daily) or placebo (*n* = 2991). The population was divided into resHTN (*n* = 1406), uctrHTN (*n* = 581), and ctrHTN (*n* = 3546) (i.e. normal SBP, hypertension, or few with no hypertension as 90% having a history of hypertension). The flow of the analysis is depicted in Supplementary data online, *Figure S1*. *Table 1* presents the baseline characteristics across hypertension categories. There were some significant differences in age, race, region, and ejection fraction. Patients with resHTN had slightly higher weight and body mass index. While eGFR showed no significant differences, elevated urine albumin excretion was significantly more prominent in patients with resHTN than ctrHTN and uctrHTN. Resistant hypertension was more frequently associated with diabetes. Mineralocorticoid receptor antagonist use was more prevalent in ctrHTN vs. resHTN (39.7% vs. 35.7%), and β-blocker use was more common in resHTN than ctrHTN (93.3% vs. 80.7%). *Table 1* (right side) summarizes the same data for patients with resHTN without MRA or with MRA.

**Table 1:** Baseline characteristics of the study population and subgroups

	Resistant hypertension (*n* = 1406)		Uncontrolled hypertension (*n* = 581)		Controlled hypertension (*n* = 3546)		*P*-value	With MRA (*n* = 502)		Without MRA (*n* = 904)	
**SBP (mmHg)**	149.6	(9.5)	147.1	(7.7)	125.9	(8.3)	NA	149.0	(9.2)	149.9	(9.7)
**HR (b.p.m.)**	69.4	(11.6)	69.6	(11.7)	70.7	(11.9)	.0010	70.5	(11.8)	68.8	(11.4)
**Sex**							.2059				
** Female**	655	(46.6)	255	(43.9)	1555	(43.9)		216	(43.0)	439	(48.6)
** Male**	751	(53.4)	326	(56.1)	1991	(56.1)		286	(57.0)	465	(51.4)
**Age (years)**	72.3	(9.2)	73.9	(8.4)	71.6	(9.5)	<.0001	70.4	(9.5)	73.3	(8.8)
**Race**							<.0001				
** White**	1087	(77.3)	415	(71.4)	2731	(77.0)		385	(76.7)	702	(77.7)
** Black/African-American**	90	(6.4)	23	(4.0)	124	(3.5)		26	(5.2)	64	(7.1)
** Asian**	130	(9.2)	109	(18.8)	489	(13.8)		58	(11.6)	72	(8.0)
** Other incl. mixed**	99	(7.0)	34	(5.9)	200	(5.6)		33	(6.6)	66	(7.3)
**Region**							<.0001				
** North America**	138	(9.8)	82	(14.1)	417	(11.8)		26	(5.2)	112	(12.4)
** Latin America**	322	(22.9)	115	(19.8)	950	(26.8)		138	(27.5)	184	(20.4)
** Europe**	748	(53.2)	254	(43.7)	1561	(44.0)		263	(52.4)	485	(53.7)
** Asia**	112	(8.0)	81	(13.9)	405	(11.4)		52	(10.4)	60	(6.6)
** Other**	86	(6.1)	49	(8.4)	213	(6.0)		23	(4.6)	63	(7.0)
**LVEF (%)**	54.4	(8.8)	55.5	(8.5)	54.1	(8.8)	.0020	53.0	(8.7)	55.3	(8.7)
**NT-proBNP (pg/mL)**	933	(482–1685)	919	(499–1571)	979	(500–1739)	.5484*	905	(470–1677)	946	(486–1686)
**HS troponin T (ng/L)**	18.7	(12.7–27.6)	17.2	(11.5–26.1)	17.4	(11.5–26.6)	.0168*	18.4	(12.0–26.3)	18.7	(13.0–28.5)
**Weight (kg)**	84.9	(19.5)	78.9	(18.9)	81.6	(19.3)	<.0001	85.2	(19.5)	84.7	(19.6)
**BMI (kg/m^2^)**	30.9	(6.0)	28.8	(5.8)	29.7	(5.8)	<.0001	30.9	(6.0)	31.0	(6.0)
**eGFR (mL/min/1.73 m^2^)**	60.1	(19.8)	60.9	(18.8)	60.9	(19.8)	.4423	62.8	(19.7)	58.6	(19.7)
** <60**	714	(50.8)	277	(47.7)	1767	(49.8)	.4524	232	(46.2)	482	(53.3)
**UACR (mg/g)**							<.0001				
** <30**	684	(48.6)	312	(53.7)	2178	(61.4)		258	(51.4)	426	(47.1)
** 30–300**	457	(32.5)	190	(32.7)	1077	(30.4)		153	(30.5)	304	(33.6)
** >300**	261	(18.6)	74	(12.7)	276	(7.8)		88	(17.5)	173	(19.1)
**Haemoglobin (g/dL)**	13.3	(1.6)	13.4	(1.6)	13.3	(1.6)	.5425	13.5	(1.6)	13.2	(1.6)
**History of Afib/flutter**	686	(48.8)	283	(48.7)	1896	(53.5)	.0028	244	(48.6)	442	(48.9)
**History of HHF**	324	(23.0)	100	(17.2)	821	(23.2)	.0055	146	(29.1)	178	(19.7)
**NYHA Class I/II**	1106	(78.7)	487	(83.8)	2948	(83.1)	.0005	400	(79.7)	706	(78.1)
**NYHA Class III/IV**	300	(21.3)	94	(16.2)	598	(16.9)		102	(20.3)	198	(21.9)
**Cause of HF**							.6550				
** Ischsemic**	509	(36.2)	215	(37.0)	1251	(35.3)		211	(42.0)	298	(33.0
** Non-ischaemic**	897	(63.8)	366	(63.0)	2294	(64.7)		291	(58.0)	606	(67.0)
**DM**	813	(57.8)	247	(42.5)	1684	(47.5)	<.0001	302	(60.2)	511	(56.5)
**ACEi/ARB/ARNi**	1331	(94.7)	345	(59.4)	2809	(79.2)	<.0001	483	(96.2)	848	(93.8)
**Beta-blockers**	1312	(93.3)	376	(64.7)	3077	(86.8)	<.0001	474	(94.4)	838	(92.7)
**Diuretics (other than MRA)**	1309	(93.1)	260	(44.8)	2863	(80.7)	<.0001	405	(80.7)	904	(100.0)
**MRA**	502	(35.7)	99	(17.0)	1409	(39.7)	<.0001	502	(100.0)	0	

Statistical tests were done with ANOVA for continuous variables and *χ*^2^ for categorical variables. *N-terminal pro-B-type natriuretic peptide and HB troponin were based on log transformed results.

Data are presented as mean (SD) or median (*Q*1–*Q*3).

ACEi, angiotensin-converting enzyme inhibitor; Afib, atrial fibrillation; ARB, angiotensin receptor blocker; ARNi, angiotensin receptor–neprilysin inhibitor; BMI, body mass index; DM, diabetes mellitus; eGFR, estimated glomerular filtration rate; HF, heart failure; HHF, hospitalization for heart failure; HR, heart rate; HS, high sensitive; LVEF, left ventricular ejection fraction; MRA, mineralocorticoid receptor antagonist; NT-proBNP, N-terminal pro-B-type natriuretic peptide; NYHA, New York Heart Association; SBP, systolic blood pressure; UACR, urine albumin–creatinine ratio.

### Association of hypertension categories with outcomes

Association of hypertension category with the primary composite outcome, its components (HFH or CVD), and first and recurrent HFH or all-cause death were studied in patients on placebo. The data are shown in *Figure 1*. Compared with ctrHTN (reference), the primary endpoint was more common in resHTN with a hazard ratio of 1.24 (1.01–1.53) (*Figure 1A*), while for its components, there were no significant differences. There was a significant difference between ctrHTN, uctrHTN, and resHTN (*P* = .036) for the primary endpoint, for first HFH (*P* = .003), and for first and recurrent HFH (*P* = .004) (*Figure 1C*). There was no difference between CVD (*P* = .23) and all-cause death (*P* = .21). Just evaluating resHTN treated with MRA (‘refractory’) compared with those without MRA, there was no difference in the primary outcome (*P* = .50), first HFH (*P* = .31), CVD (*P* = .64), recurrent HFH (*P* = .17), and all-cause mortality (*P* = .35) (not shown).

### Effect of empagliflozin on blood pressure by hypertension category

**Figure 2:**
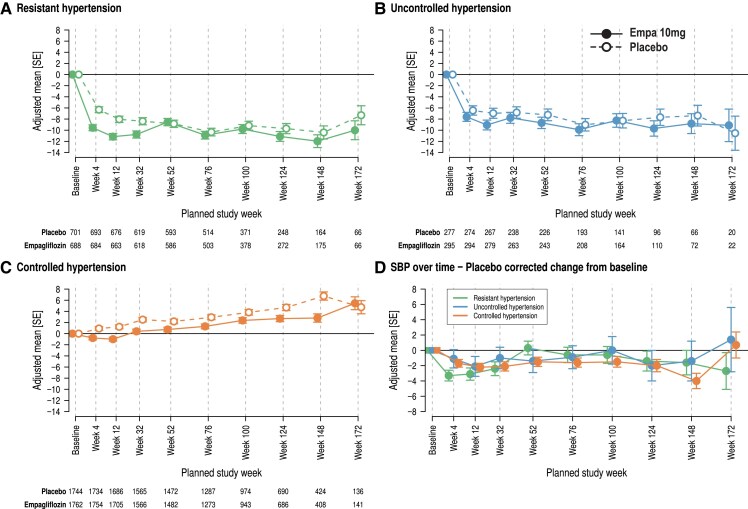
Effect of treatment with empagliflozin (filled symbols) or placebo (open symbols) in resistant hypertension (*A*), uncontrolled hypertension (*B*), and controlled hypertension (*C*) on change from baseline systolic blood pressure (*A–C*) and placebo-corrected change of systolic blood pressure over time (*D*) based on mixed model for repeated measures model adjusted for age, baseline estimated glomerular filtration rate (Chronic Kidney Disease Epidemiology Collaboration formula), baseline left ventricular ejection fraction as linear covariate(s) and region, baseline diabetes status, sex, week reachable, visit by treatment by hypertension status interaction, and baseline systolic blood pressure by visit interaction as fixed effect(s). SE, standard error; SBP, systolic blood pressure

*Figure 2* summarizes the effects of empagliflozin compared with placebo on SBP in resHTN (*Figure 2A*), uctrHTN (*Figure 2B*), and ctrHTN (*Figure 2C*) as well as the placebo-corrected change in the three groups (*Figure 2D*). In patients with resHTN and uctrHTN, SBP drops on placebo and on empagliflozin with some differences between empagliflozin and placebo in resHTN but no significant differences in uctrHTN. Over time, BP increased in patients with ctrHTN (*Figure 2C*) on placebo and empagliflozin with a lower extent on empagliflozin (*P* < .0001–.04 until Week 144). The baseline SBP was expectedly lower in ctrHTN than in resHTN and uctrHTN. The interaction *P*-values were between .15 and .92. In resHTN at Weeks 4–32, there was a significant treatment difference (*P* = .001–.009) with a mean difference in SBP between 2.4 and 3.3 mmHg, while later from Weeks 52–172, BP values were similar (*P* = .26–.74). Placebo-corrected SBP changes by empagliflozin are shown in *Figure 2D*. In resHTN, the placebo-corrected SBP change from baseline by empagliflozin was not different with or without MRA treatment (see Supplementary data online, *Figure S2*).

**Figure 3:**
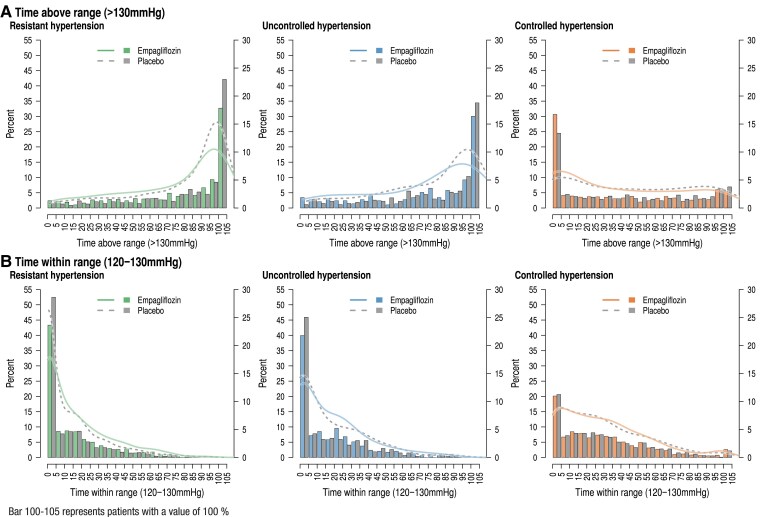
Time above range (>130 mmHg) (*A*) and time within range (120–130 mmHg) (*B*) in resistant hypertension (left), uncontrolled hypertension (middle), and controlled hypertension (right). Population densities are given for empagliflozin (solid line) and placebo (broken line) with percentage categories of patients being above range on placebo (left bar) and on empagliflozin (right bar) given in the right ordinate as well as time within range for the same groups

To have a more sensitive approach to detect empagliflozin’s effect on SBP in hypertension phenotypes, we explored the time above range (>130 mmHg) (*Figure 3A*) as well as the time in range (120–130 mmHg) (*Figure 3B*) in resHTN, uctrHTN, and ctrHTN. In resHTN and uctrHTN, more patients on placebo were for a longer period out of therapeutic range. In controlled hypertension (*Figure 3A*), more patients had very small times above range on empagliflozin and fewer patients had some time points above range than on placebo. In resHTN and ctrHTN, altogether, the time in target range was increased by empagliflozin while there was no meaningful difference in ctrHTN (*Figure 3B*). We observed some shifts from ctrHTN and uctrHTN on placebo (5.8%) and on empagliflozin (4.9%) and from ctrHTN to resHTN with 11.9% on placebo and 9.3% on empagliflozin. Patients with resHTN changed to ctrHTN or SBP < 110 mmHg in 11.7% on placebo and 12.2% on empagliflozin. The shift was determined by comparing the baseline and the last value on treatment of SBP measurement.

### Effect of empagliflozin on incident hypertensive urgencies

**Figure 4:**
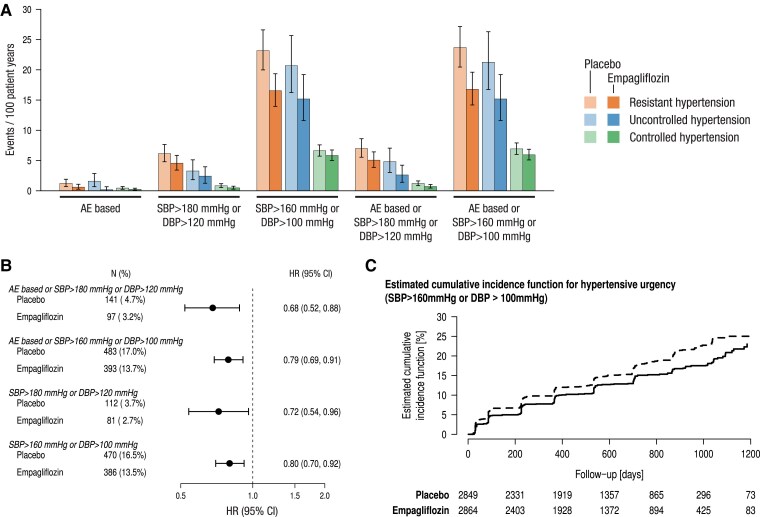
Incidence rates (*A*) (events per 100 patient years), treatment effect of empagliflozin compared with placebo (*B*), and estimated cumulative incidence function (*C*) for hypertensive urgencies according to different criteria. Treatment effect based on Cox regression model adjusted for age, baseline estimated glomerular filtration rate (Chronic Kidney Disease Epidemiology Collaboration formula), baseline left ventricular ejection fraction as linear covariates and region, baseline diabetes status, and sex. Patients with increased blood pressure values at baseline were not at risk for occurrence of the respective endpoint. AE, adverse event; CI, confidence interval; DBP; diastolic blood pressure; HR, hazard ratio; SBP, systolic blood pressure

Incident hypertensive urgencies were explored using different definition criteria: pure adverse event-based reporting and criteria of visit SBP > 180 mmHg or DBP > 120 mmHg, SBP > 160 mmHg or DBP > 100 mmHg or the combination of the BP criteria with adverse event reporting. *Figure 4A* shows 20%–32% reductions of incident hypertensive urgencies with empagliflozin (*Figure 4B*) with different prevalence according to the used criteria. A decrease in incident hypertensive urgencies was reported throughout the study (*Figure 4C*).

### Effect of empagliflozin on outcomes by hypertension categories

**Figure 5:**
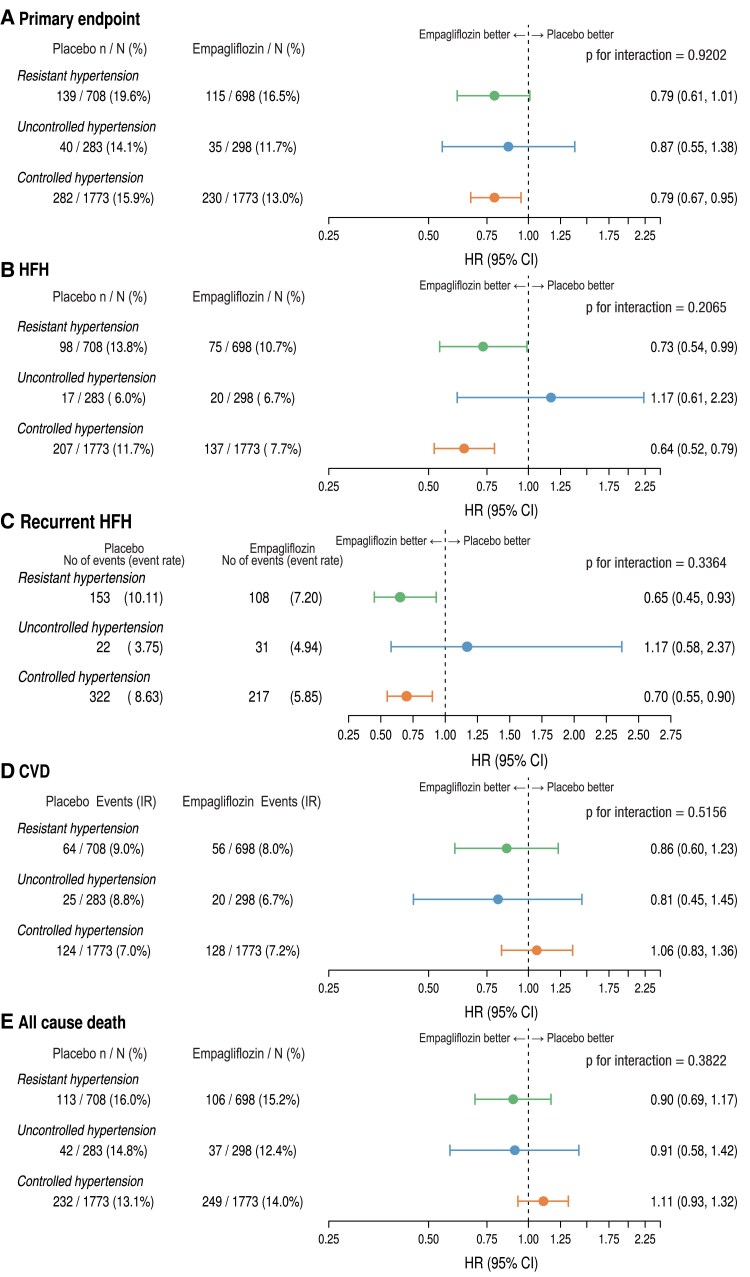
Effect of empagliflozin vs. placebo on the primary endpoint (*A*), heart failure hospitalization (*B*), recurrent heart failure hospitalization (*C*), cardiovascular death (*D*), and all-cause death (*E*). Cox regression models were examined using prespecified covariates of age, sex, geographical region, diabetes status at baseline, left ventricular ejection fraction, eGFR at baseline, hypertension category and, hypertension category ∗ treatment interaction. CI, confidence intervals; CVD, cardiovascular death; HFH, heart failure hospitalization; HR, hazard ratio

*Figure 5* shows forest plots of the effect of empagliflozin on the primary endpoint, HFH, CVD, recurrent HFH, and all-cause death across the hypertensive categories. There was no significant interaction between the treatment effects of empagliflozin concerning all studied endpoints. The cumulative incidence curves are shown in Supplementary data online, *Figure S3*. By further dividing resHTN in those without MRA and those treated with MRA, there was also no significant difference between all outcomes [*P* = .26 for primary endpoint, *P* = .38 for first HFH, *P* = .67 for recurrent HFH, *P* = .55 for CVD, and *P* = .82 for all-cause death (not shown)].

### Effect of empagliflozin on kidney function according to hypertension category

**Figure 6:**
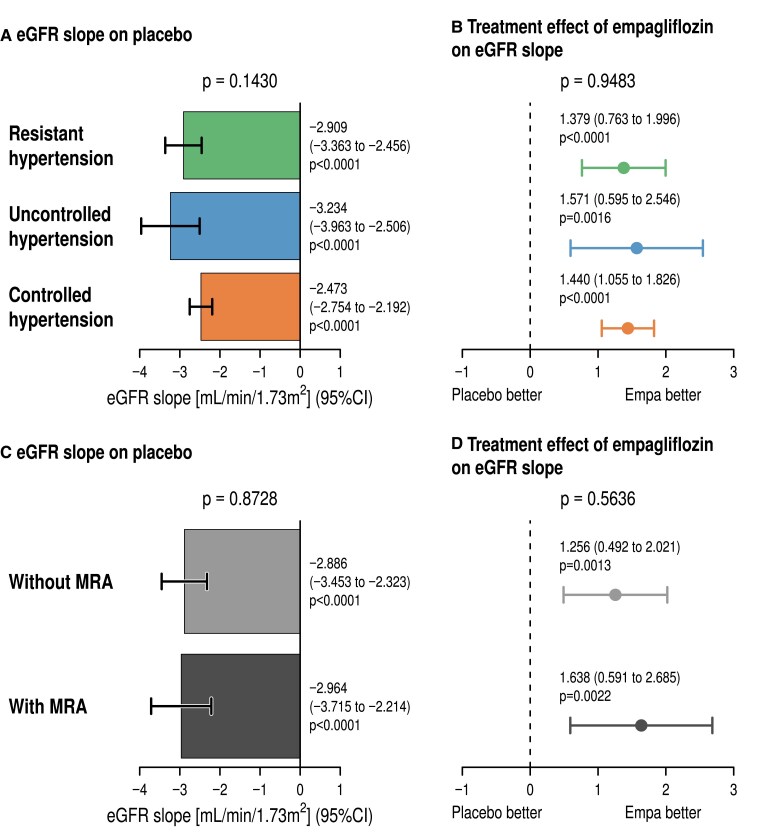
Estimated glomerular filtration rate chronic slopes within placebo (*A*) in resistant hypertension, uncontrolled hypertension, and controlled hypertension and treatment effect of empagliflozin (*B*) in resistant hypertension, uncontrolled hypertension, and controlled hypertension. Estimated glomerular filtration rate slopes in resistant hypertension with and without mineralocorticoid receptor antagonist treatment (*C*) and treatment effect of empagliflozin on estimated glomerular filtration rate slopes in patients treated without (above) and with mineralocorticoid receptor antagonist. CI, confidence interval; eGFR, estimated glomerular filtration rate; MRA, mineralocorticoid receptor antagonist

*Figure 6* summarizes the effects of the hypertension categories on the chronic eGFR slope (*Figure 6A*) and the treatment effect of empagliflozin (*Figure 6B*). On placebo, there was no difference in the eGFR slopes over time between the hypertension categories with a numerically slower eGFR decline in ctrHTN resulting in a *P*-value of .143. The treatment effect of empagliflozin between resHTN, ctrHTN, and uctrHTN was not different with a *P*-value for interaction of .95. There was also no difference in the eGFR slope on placebo for patients treated with MRA (‘refractory’) or without MRA (*P* = .87) (*Figure 6C*) and also no interaction with the treatment effect of empagliflozin on the eGFR slope (*P* = .56) (*Figure 6D*). There was also no significant interaction of KCCQ-CSS between hypertension phenotype and no differences between the treatment effects of empagliflozin on KCCQ-CSS until Week 52 (see Supplementary data online, *Figure S4*).

### Adverse events by hypertension category

The incidence of any adverse events as well as events leading to discontinuation was similar between empagliflozin and placebo in all hypertension categories. There was also no difference of effect between the hypertension categories observed on the incidence rates of adverse safety events on placebo or empagliflozin indicating that adverse events were generally balanced between the treatment arms and across hypertension categories (see Supplementary data online, *Table S1*).

## Discussion

Hypertension is the most prevalent risk factor for HFpEF^4,5^ and it remains in the later stages one of the most important comorbidities further augmenting left ventricular hypertrophy, diastolic dysfunction, arterial ventricular uncoupling, and other complications such as renal insufficiency, further accelerating the course of the syndrome.^4^ In this study, we observed resHTN in 23.5% of the overall EMPEROR population, while 59.2% had normal BP values and 9.7% uncontrolled but not resistant, i.e. receiving less than three antihypertensive drug classes. Compared with ctrHTN, there was a 24% increase of the primary outcome HFH and CVD, while no significant differences occurred for the components of the primary outcome, recurrent HFH, and cardiovascular or all-cause death. In uctrHTN and resHTN, eGFR decline as a surrogate for the rate of kidney disease progression was only numerically but not significantly greater than in ctrHTN. The treatment effect of empagliflozin on the primary composite outcome as well as its components, first and recurrent HFH, and KCCQ-CSS or eGFR slope were similar. Empagliflozin had only minor effects on BP in uctrHTN and resHTN but slightly reduced the time above range of treatment goals in patients ≥80% above range in resHTN and uctrHTN but not in ctrHTN and increased the number of patients within range in resHTN and uctrHTN. Empagliflozin also reduced incident hypertensive urgencies. Overall, the treatment effects of empagliflozin were not affected by hypertension categories or MRA treatment in resHTN.

Resistant hypertension is defined as a BP above target despite the use of at least three antihypertensive drugs of different classes, one being a diuretic.^5–9,12^ Herein, we used the 2018 ESC^17^ and 2023 ESH^7^ guideline definition defining a SBP > 140 mmHg as uncontrolled. In patients with hypertension, the prevalence of apparent resHTN was 10%–15% with a range of 5%–35%. Applying the American College of Cardiology/American Heart Association guidelines with lower boundaries (≥130 mmHg),^6^ the prevalence of resHTN rises from 7.5% to 14% in the ACCORD trial.^18^ For treatment targets, we used a stricter definition with the ESC/ESH treatment target window of 120–130 mmHg.^17^ As in HFpEF^18–21^ and in HFrEF,^22–24^ there is a U- or J-shaped curve in the clinical trials but also in registries,^25^ and patients with a SBP < 110 mmHg were excluded from this analysis as this effect is potentially due to inverse causation with rising risk at a low SBP.^19^ Moreover, this study aimed to deal with high SBP in HFpEF.

Herein, we observed a slight increase in the primary composite outcomes and HFH in resHTN compared with ctrHTN. This was not observed for CVD and all-cause death. We found in resHTN more patients with diabetes mellitus and hypertension-related end-organ damage including chronic kidney disease. Based on the outcomes of the PATHWAY-2 trial,^26^ guidelines recommend treatment of resHTN with spironolactone as fourth-line antihypertensive agent.^7,17^ We looked at patients treated with resHTN on spironolactone who were still uncontrolled (sometimes referred to as ‘refractory’ hypertension) and found no significant differences between spironolactone and no spironolactone treatment in cardiovascular outcomes, eGFR slope, and quality of life in the placebo group. These findings align with the recent analysis from DELIVER, where also no significant differences in outcomes with dapagliflozin were observed.^27^

Herein, empagliflozin only slightly reduced SBP. There was an overall increase of SBP in ctrHTN and a decline in uctrHTN and resHTN over time, likely related to regression to the mean. The placebo-corrected change of SBP by empagliflozin was minor. This is in agreement with data from EMPEROR-Preserved,^20^ EMPA-REG OUTCOME^28^ as well as EMPEROR-Reduced,^24^ DAPA-HF,^29^ and DELIVER,^21^ where, at low BP, no significant drop in BP was observed. In DELIVER, there was no better BP control rate by dapagliflozin in patients with resHTN.^30^ Herein, we looked at a more sensitive method of time above target range and time in target range^31^ closely associated with cardiovascular and renal outcomes.^32^ Some changes were observed towards a better BP control, but the meaning of these findings remains unknown as in these patients, there was no reduction of cardiovascular outcome rates, renal outcomes, or quality of life. Altogether, these data provide evidence that small modifications of SBP, a lack of improved BP control rates, and the minor shifts of some patients into higher time in target ranges as well as the previously observed slight reduction of central BP do not play a significant role in the outcome effects of empagliflozin. Nevertheless, there was a significant reduction in hypertensive urgencies, when different definitions were used. Empagliflozin treatment might have an impact on patients with particularly high SBP by reducing these events. Although hypertensive urgencies are strongly associated with outcomes,^32^ the effect of empagliflozin on hypertension urgencies compared with placebo did not modify outcomes in the overall HFpEF population of EMPEROR-Preserved.

Empagliflozin reduced CVD and HFH and reduced eGFR slope while improving quality of life in patients with HFpEF^13^ irrespective of SBP.^20^ Separating the overall population of EMPEROR-Preserved in different hypertension categories did not modify the cardio-renal effects of empagliflozin. The impact of empagliflozin to increase patients’ time in target range and reduce time above range might not contribute mechanistically to the treatment effect of empagliflozin on CVD and HFH, kidney outcomes, and quality of life. We extend those findings to patients with MRA-treated and MRA-naïve resHTN showing no impact of spironolactone. In patients with hypertension, resHTN has been related to sodium intake and overload, and as SGLT2 inhibitors lead to a modest increase of sodium excretion, some differences could have been expected by empagliflozin. However, there were no different effects on SBP by empagliflozin. Furthermore, the magnitude of benefit on SBP reduction and clinical outcomes was not different between the groups, and empagliflozin unfolds similar protective effects irrespective of the hypertension categories. The data are summarized in the *Structured Graphical Abstract*.

This study could be affected by some limitations. The definition of resHTN is based on baseline SBP measurements but not on ambulatory BP recordings or home measurements as suggested by guidelines.^7,17^ Therefore, we cannot exclude the presence of white-coat hypertension in some of the EMPEROR patients. Furthermore, effects of treatment resistance related to non-adherence to medication intake (‘pseudo-resistance’), white-coat phenomenon, incorrect SBP measurement at baseline, and potential drug intake of substances increasing SBP need to be considered.^33^ Of course, toxicological drug testing to assure medication adherence was not done as it is not possible to test all accompanying treatments including those for hypertension in such a large outcome trial. However, these confounders were inevitable as ambulatory BP recordings and biochemical drug monitoring in such a large outcome trial are impossible.

## Conclusion

In EMPEROR-Preserved, the prevalence of resHTN was high and associated with the highest rates of the primary composite endpoint compared with other hypertensive categories. The treatment effect of empagliflozin was not affected, and treatment with empagliflozin was safe across the hypertensive categories. The modest reduction of BP as well as the slight increase of patients with higher rates of time in target range and less incident hypertensive urgencies apparently did not contribute to the overall beneficial effects of empagliflozin in HFpEF, which produced a similar relative but a slightly higher absolute risk reduction in patients with resHTN without being modified by guideline-directed MRA treatment.

## Supplementary Material

ehae938_Supplementary_Data

## Declarations

### Data Availability

To ensure independent interpretation of clinical study results and enable authors to fulfil their role and obligations under the ICMJE criteria, Boehringer Ingelheim grants all external authors access to relevant clinical study data. In adherence with the Boehringer Ingelheim Policy on Transparency and Publication of Clinical Study Data, scientific and medical researchers can request access to clinical study data, typically, 1 year after the approval has been granted by major Regulatory Authorities or after termination of the development program. Researchers should use the https://vivli.org/ link to request access to study data and visit https://www.mystudywindow.com/msw/datasharing for further information.

### Pre-registered Clinical Trial Number

The pre-registered clinical trial number is www.clinicaltrials.gov—unique identifier: NCT03057951.
