# Supplementary material for: Empagliflozin in resistant hypertension and heart failure with preserved ejection fraction: the EMPEROR-Preserved trial
Source: Eur Heart J. 2025 Mar 4;46(14):1304–17. doi: 10.1093/eurheartj/ehae938 (PMC11973566; doi:10.1093/eurheartj/ehae938)
Supplement: ehae938_Supplementary_Data [file ehae938_supplementary_data.pdf]

Supplement table 1 Adverse events

	Resistant Hypertension		Uncontrolled hypertension		Controlled hypertension		With MRA		Without MRA	
	Placebo N (%)	Empagloflozin N (%)	Placebo N (%)	Empagloflozin N (%)	Placebo N (%)	Empagloflozin N (%)	Placebo N (%)	Empagloflozin N (%)	Placebo N (%)	Empagloflozin N (%)
Total number of patients	708 (100.0)	697 (100.0)	282 (100.0)	298 (100.0)	1772 (100.0)	1773 (100.0)	262 (100.0)	239 (100.0)	446 (100.0)	458 (100.0)
Total with adverse events	621 (87.7)	602 (86.4)	245 (86.9)	256 (85.9)	1518 (85.7)	1505 (84.9)	233 (88.9)	197 (82.4)	388 (87.0)	405 (88.4)
Patients with adverse events leading to drug discontinuation	149 (21.0)	138 (19.8)	53 (18.8)	59 (19.8)	298 (16.8)	335 (18.9)	64 (24.4)	43 (18.0)	85 (19.1)	95 (20.7)
Adverse Events of Special Interest										
Acute renal failure	92 (13.0)	106 (15.2)	31 (11.0)	28 (9.4)	230 (13.0)	202 (11.4)	41 (15.6)	38 (15.9)	51 (11.4)	68 (14.8)
Volume depletion	61 (8.6)	87 (12.5)	19 (6.7)	25 (8.4)	172 (9.7)	201 (11.3)	26 (9.9)	25 (10.5)	35 (7.8)	62 (13.5)
Hypotension	54 (7.6)	79 (11.3)	17 (6.0)	21 (7.0)	155 (8.7)	174 (9.8)	23 (8.8)	24 (10.0)	31 (7.0)	55 (12.0)
Symptomatic hypotension	33 (4.7)	50 (7.2)	10 (3.5)	12 (4.0)	94 (5.3)	109 (6.1)	13 (5.0)	16 (6.7)	20 (4.5)	34 (7.4)

Supplement figure 1

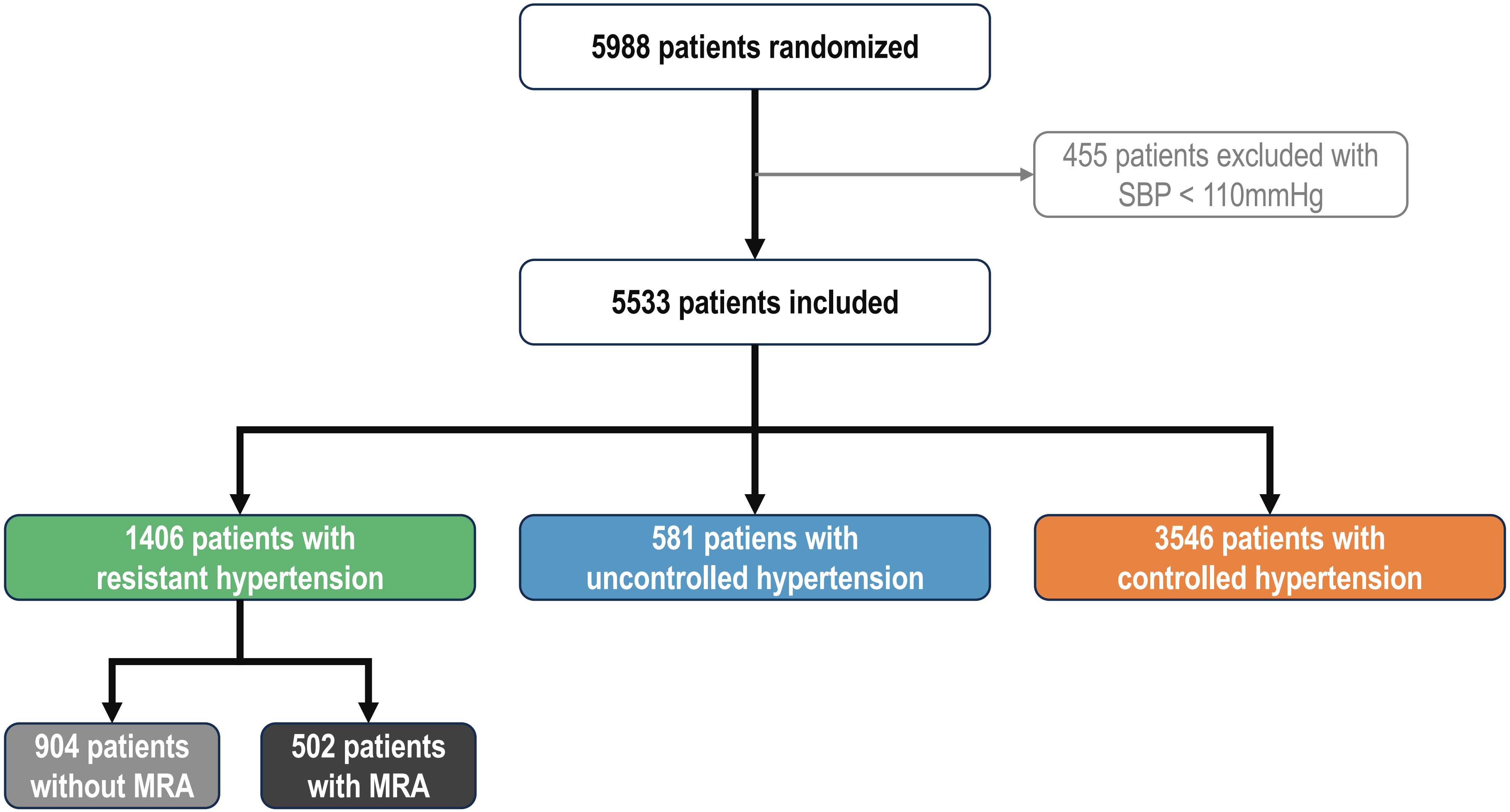

Supplement figure 2

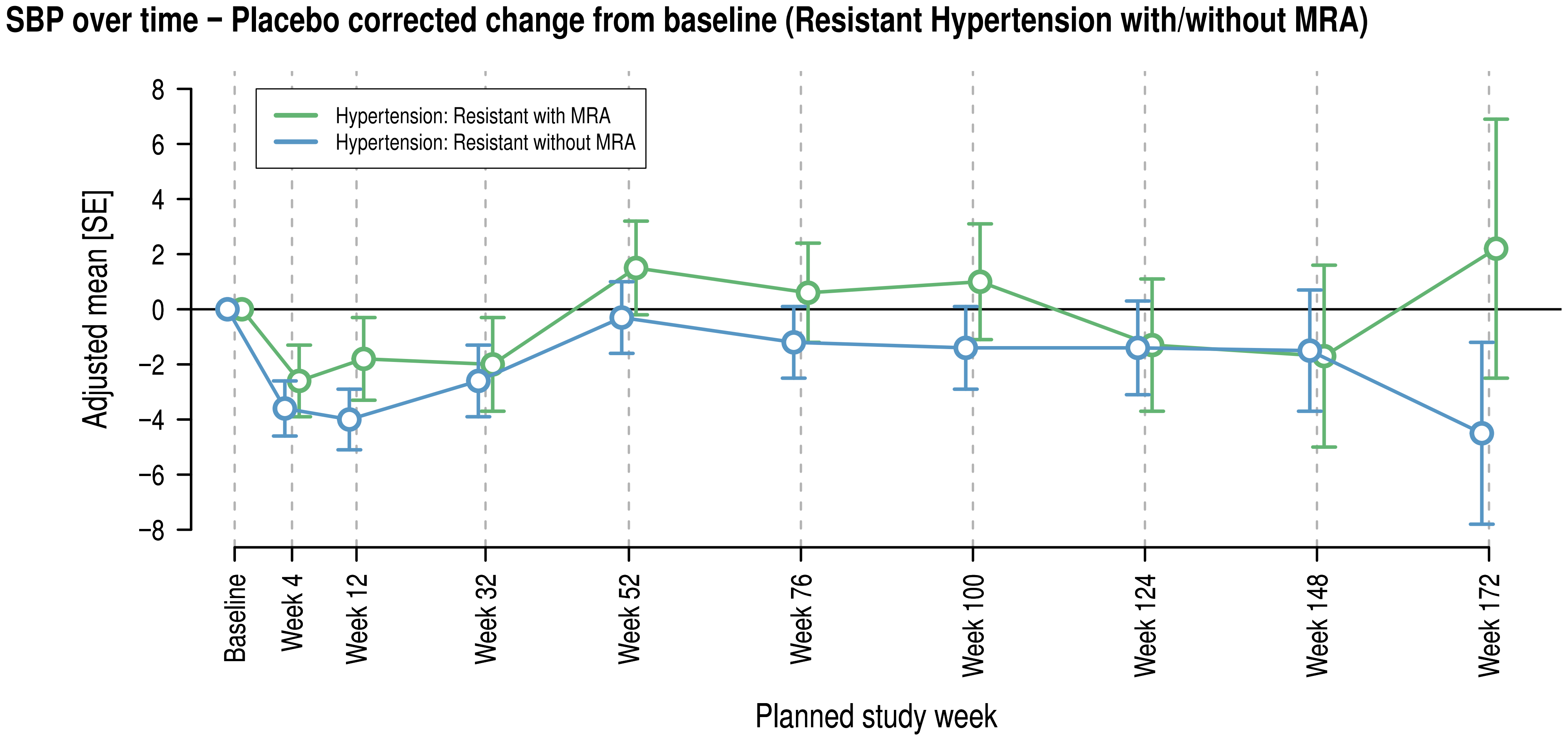

Supplement figure 3

A Primary endpoint (CVD or HHF)

Resistant hypertension | Uncontrolled hypertension | Controlled hypertension

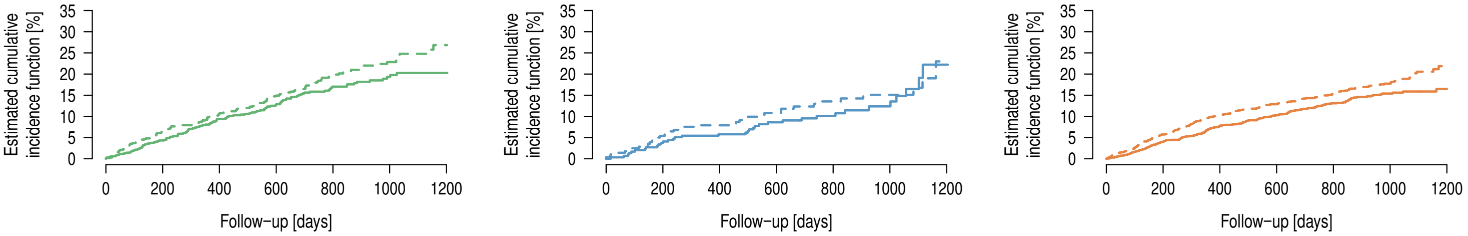

Subgroup		0	200	400	600	800	1000	1200
Resistant hypertension	Placebo	708	658	617	453	294	156	38
Resistant hypertension	Empagliflozin	698	659	608	455	323	170	37
Uncontrolled hypertension	Placebo	283	262	248	181	126	70	11
Uncontrolled hypertension	Empagliflozin	298	282	267	202	134	72	5
Controlled hypertension	Placebo	1773	1644	1539	1148	787	381	64
Controlled hypertension	Empagliflozin	1773	1676	1581	1177	796	388	85

B Hospitalisation for heart failure

Resistant hypertension | Uncontrolled hypertension | Controlled hypertension

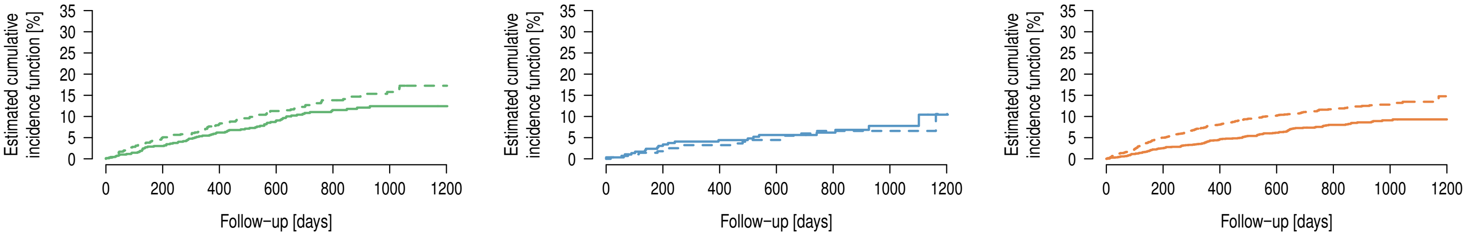

Subgroup		0	200	400	600	800	1000	1200
Resistant hypertension	Placebo	708	658	617	453	294	156	38
Resistant hypertension	Empagliflozin	698	659	608	455	323	170	37
Uncontrolled hypertension	Placebo	283	262	248	181	126	70	11
Uncontrolled hypertension	Empagliflozin	298	282	267	202	134	72	5
Controlled hypertension	Placebo	1773	1644	1539	1148	787	381	64
Controlled hypertension	Empagliflozin	1773	1676	1581	1177	796	388	85

C Recurrent Hospitalisation for heart failure

Resistant hypertension | Uncontrolled hypertension | Controlled hypertension

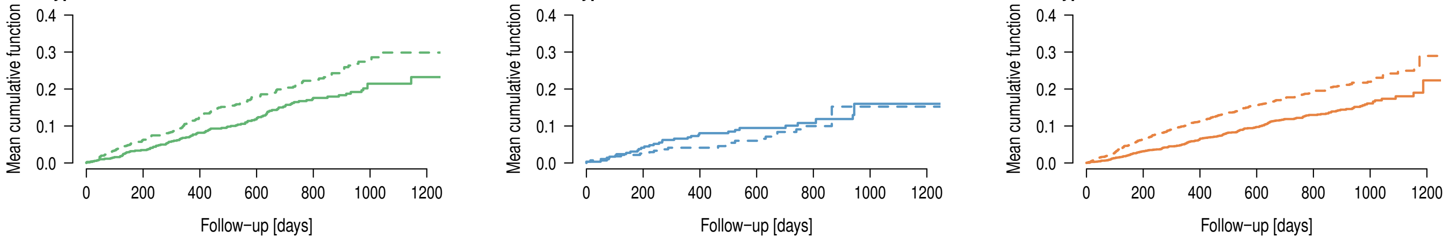

Subgroup		0	200	400	600	800	1000	1200
Resistant hypertension	Placebo	708	690	666	506	337	175	42
Resistant hypertension	Empagliflozin	698	678	641	491	363	190	42
Uncontrolled hypertension	Placebo	283	267	255	189	136	74	12
Uncontrolled hypertension	Empagliflozin	298	289	276	212	143	77	6
Controlled hypertension	Placebo	1773	1720	1658	1256	865	419	72
Controlled hypertension	Empagliflozin	1773	1713	1643	1238	841	415	88

D Cardiovascular death

Resistant hypertension | Uncontrolled hypertension | Controlled hypertension

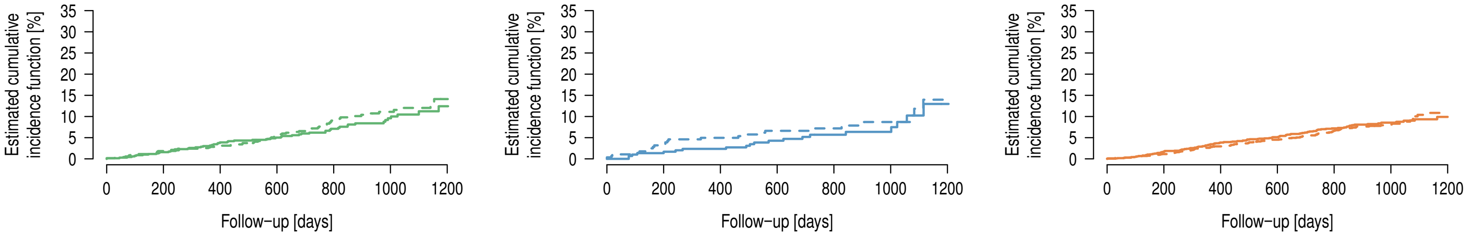

Subgroup		0	200	400	600	800	1000	1200
Resistant hypertension	Placebo	708	693	669	510	344	183	43
Resistant hypertension	Empagliflozin	698	681	649	502	372	196	43
Uncontrolled hypertension	Placebo	283	272	262	194	140	76	12
Uncontrolled hypertension	Empagliflozin	298	292	281	218	146	79	6
Controlled hypertension	Placebo	1773	1733	1681	1290	895	440	78
Controlled hypertension	Empagliflozin	1773	1720	1659	1260	865	432	92

E All cause death

Resistant hypertension | Uncontrolled hypertension | Controlled hypertension

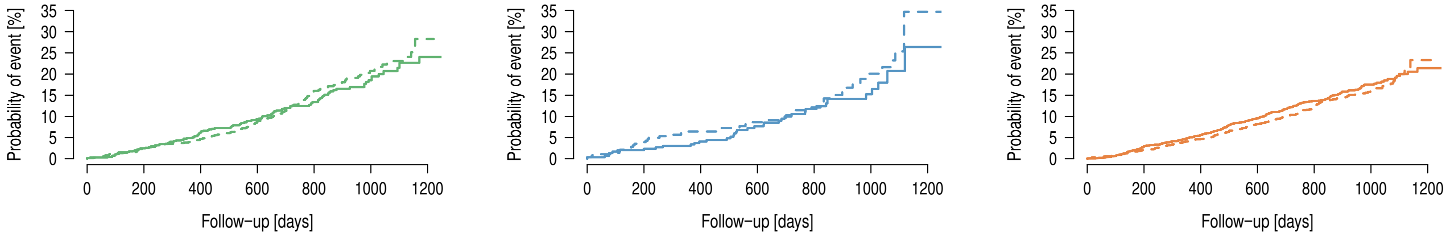

Subgroup		0	200	400	600	800	1000	1200
Resistant hypertension	Placebo	708	693	669	510	344	183	43
Resistant hypertension	Empagliflozin	698	681	649	502	372	196	43
Uncontrolled hypertension	Placebo	283	272	262	194	140	76	12
Uncontrolled hypertension	Empagliflozin	298	292	281	218	146	79	6
Controlled hypertension	Placebo	1773	1733	1681	1290	895	440	78
Controlled hypertension	Empagliflozin	1773	1720	1659	1260	865	432	92

Legend: —— Empa 10mg; - - - - Placebo

Supplement figure 4

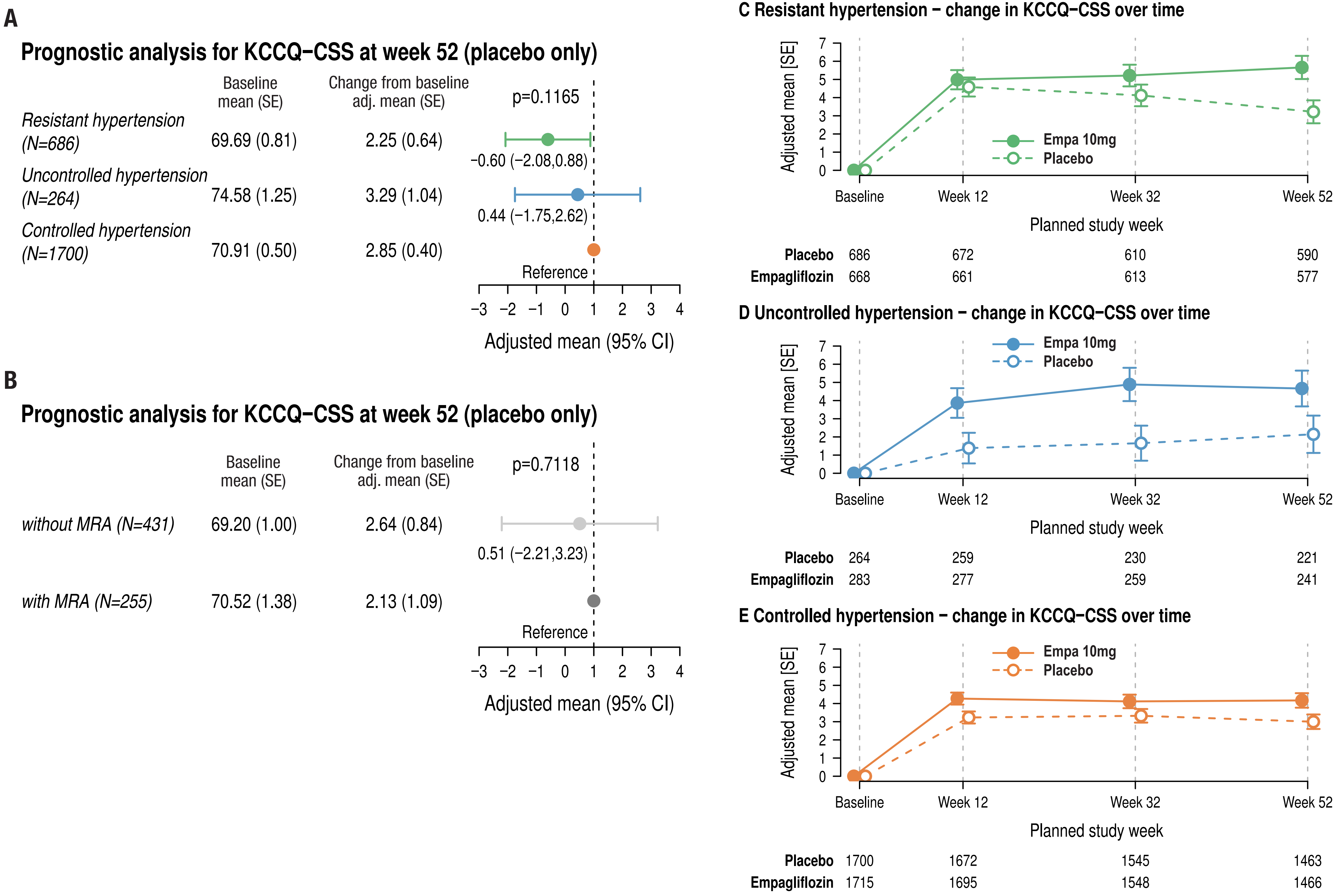

Supplement Figure 1

Patient flow of the present analysis.

MRA: mineralocorticoid receptor antagonist; SBP: systolic blood pressure

Supplement Figure 2

Placebo-corrected systolic blood pressure (SBP) change from baseline in patients treated with MRA (green) and without MRA (blue) in resistant hypertension.

Supplement Figure 3

Cumulative incidence adjusting for CV-death or all-cause death or Kaplan Meier (for all-cause death) curves in patients treated with empagliflozin 10 mg (solid line) or placebo (dashed line) for the primary endpoint (A), heart failure hospitalization (HFH, B), recurrent HFH (C), CVD (D) and all-cause death (E) in resistant hypertension (resHTN, green, left), uncontrolled hypertension (uctrHTN, blue, middle) and controlled hypertension (ctrHTN, red, right).

Supplement Figure 4

Baseline and change from baseline to week 52 in Kansas City Cardiomyopathy Questionnaire-Clinical Summary Score (KCCQ-CSS) in patients on placebo with resistant hypertension (resHTN), uncontrolled hypertension (uctrHTN) and controlled hypertension (ctrHTN) (A) as well as in resHTN patients treated without or with MRA (B).

Adjusted mean change in Kansas City Cardiomyopathy Questionnaire-Clinical Summary Score (KCCQ-CSS) in resistant hypertension (resHTN, A), uncontrolled hypertension (uctrHTN, B) and controlled hypertension (ctrHTN, C) by empagliflozin (filled symbols) and placebo (open symbols) in hypertensive categories (C-E) based on MMRM model.

KQQC-CSS: Kansas City Cardiomyopathy Questionnaire-Clinical Summary Score;

MRA: mineralocorticoid receptor antagonist
